# QTL mapping for resistance to and tolerance for the rice root-knot nematode, *Meloidogyne graminicola*

**DOI:** 10.1186/s12863-018-0656-1

**Published:** 2018-08-06

**Authors:** Judith Galeng-Lawilao, Arvind Kumar, Dirk De Waele

**Affiliations:** 1 Laboratory of Tropical Crop Improvement, Department of Biosystems, Faculty of Bioscience Engineering, University of Leuven, Willem de Croylaan 42, 3001 Leuven, Belgium; 2 International Rice Research Rice Institute (IRRI), Dapo Box 7777, Metro Manila, Philippines; 3 Unit for Environmental Sciences and Management, North-West University, Private Bag X6001, Potchefstroom, 2520 South Africa; 4 Department of Plant Pathology, College of Agriculture, Benguet State University, La Trinidad, Benguet Philippines

**Keywords:** Asian rice, Breeding, *Meloidogyne graminicola*, *Oryza sativa*, QTLs resistance, Rice root-knot nematode, Tolerance

## Abstract

**Background:**

The root-knot nematode *Meloidogyne graminicola* is an obligate biotrophic pathogen considered to be the most damaging nematode species that causes significant yield losses to upland and rainfed lowland rice production in South and Southeast Asia. Mapping and identification of quantitative trait loci (QTL) for resistance to and tolerance for *M. graminicola* may offer a safe and economic management option to farmers. In this study, resistance to and tolerance for *M. graminicola* in Asian rice (*Oryza sativa* L.) were studied in a mapping population consisting of 300 recombinant inbred lines (RILs) derived from IR78877–208-B-1-2, an aerobic rice genotype with improved resistance to and tolerance for *M. graminicola*, and IR64, a popular, high-yielding rice mega-variety susceptible to *M. graminicola*. RILs were phenotyped for resistance and tolerance in the dry seasons of 2012 and 2013. QTL analysis was performed using 131 single nucleotide polymorphism (SNP) and 33 simple sequence repeat (SSR) markers.

**Results:**

Three QTLs with main effects on chromosomes 4 (*qMGR*_*4.1*_), 7 (*qMGR*_*7.1*_) and 9 (*qMGR*_*9.1*_) and two epistatic interactions (*qMGR*_*3.1*_/ *qMGR*_*11.1*_ and *qMGR*_*4.2*_/ *qMGR*_*8.*1_) associated with nematode reproduction that were consistent in the two seasons were detected. A QTL affecting root galling was found on chromosomes 4 (*qGR*_*4.1*_) and 8 (*qGR*_*8.1*_), and QTLs for nematode tolerance were found on chromosomes 5 (*qYR*_*5.1*_) and 11 (*qYR*_*11.1*_). These QTLs were consistent in both seasons. A QTL for grain yield was found on chromosome 10 (*qGYLD*_*10.1*_), a QTL affecting filled grains per panicle was detected on chromosome 11 (*qFG*_*11.1*_) and a QTL for fresh root weight was found on chromosomes 2 (*qFRWt*_*2.1*_), 8 (*qFRWt*_*8.1*_) and 12 (*qFRWt*_*12.1*_) in both seasons. The donor of the alleles for *qMGR*_*4.1*_, *qMGR*_*7.1*_, *qMGR*_*9.1*_, *qGR*_*4.1*_, *qGR*_*8.1*_, *qYR*_*5.1*_ and *qFRWt*_*2.1*_ was IR78877–208-B-1-2, whereas for *qYR*_*11.1*_, *qGYLD*_*10.1*_ and *qFG*_*11.1*_, *qFRWt*_*8.1*_ and *qFRWt*_*12.1*_ was IR64. Lines having favorable alleles for resistance, tolerance and yield provided better yield under nematode-infested conditions and could be a starting point of marker-assisted breeding (MAB) for the improvement of *M. graminicola* resistance and tolerance in Asian rice.

**Conclusion:**

This study identified a total of 12 QTLs with main effects and two epistatic interactions in the 1st season and 2nd season related to *M. graminicola* resistance and tolerance, and other agronomic traits such as plant yield, percentage of filled grains, and fresh and dry root weight. Rice genotypes that have the favorable alleles for resistance (*qMGR*_*4.1*_, *qMGR*_*7.1*_, *qMGR*_*9.1*_, *qGR*_*4.1*_, *qGR*_*8.1*_) and tolerance (*qYR*_*5.1*_, and *qYR*_*11.1*_,) QTLs, and which are either resistant or partially resistant and tolerant, were also selected. These selected genotypes and the identified QTLs are vital information in designing MAB for the improvement of high-yielding rice genotypes but are susceptible to *M. graminicola* infection.

**Electronic supplementary material:**

The online version of this article (10.1186/s12863-018-0656-1) contains supplementary material, which is available to authorized users.

## Background

The rice root-knot nematode, *Meloidogyne graminicola* Golden & Birchfield, has emerged as one of the most important biotrophic pathogens that can cause substantial yield losses to the production of rice in Asia [1]. This endoparasitic sedentary nematode species occurs in all South and Southeast Asian rice-producing countries surveyed so far [2]. It can be found in a wide range of rice-based production systems, including lowland as well as upland, irrigated as well as rainfed, and deepwater rice [3–7]. Economic yield losses due to *M. graminicola* have been documented for upland, lowland and deepwater rice [3, 5, 8, 9]. Recently, this nematode species was identified as one of the important soil pathogens that limit the yield of aerobic rice [10, 11].

Continuous flooding, crop rotation and the use of nematicides are the most common practices applied in the field to mitigate *M. graminicola* yield losses*.* Continuous flooding can effectively reduce nematode populations in the soil inter alia by curbing infective second-stage juveniles (J2) from invading rice roots. However, the increasing scarcity of water available for agricultural use, especially in South and Southeast Asia [12], also increasingly limits the feasibility of this practice in the field [1]. Crop rotation with poor or non-hosts of *M. graminicola*, such as mung bean, mustard and sesame [13, 14], can also effectively reduce the population densities of *M. graminicola* in the soil, thereby reducing yield losses. However, shifting to another crop, albeit for only part of the crop season, may come with an unacceptable cost for many small-scale rice farmers in Asia, where rice is the staple food. While the use of nematicides may guarantee to some degree the control of *M. graminicola*, this practice does not offer a feasible option, especially for small-scale farmers, because these chemicals are expensive and often harmful to the environment. Moreover, most of the chemicals for nematode control, such as DBCP (1, 2-di bromo-3 chloropropane) and EDB (ethylene di-bromide), are already banned from the market [15] or are in the process of being banned. In this context, growing resistant or tolerant rice varieties may offer an effective, economic and environmentally acceptable practice in keeping *M. graminicola* population densities below economically damaging threshold levels. The significance of developing *M. graminicola*-resistant or -tolerant rice varieties will increase with rice cultivation practices that are likely to shift from prolonged flooding to more water-saving practices because of decreased water availability as a result of climate change, higher labour costs and urbanisation [1, 12, 16]. The use of these water-saving practices favours the penetration and build-up of high population densities of *M. graminicola* in the roots of susceptible rice varieties, resulting in greater damage and higher yield loss [17–19].

Resistance to *M. graminicola* has been found in *Oryza longistaminata* A. Chev. & Roehrich [20], in African rice (*O. glaberrima* Steud.) [20–22] and also in Asian rice (*Oryza sativa* L.) [23–27]. However, few of these so-called resistant Asian rice varieties are truly resistant and majority of the Asian rice germplasm is susceptible to *M. graminicola* ([7]; De Waele, personal communication). Efforts have been made to introgress resistance to *M. graminicola* from African rice into Asian rice, but this without much success as the interspecific progenies did not express the same degree of resistance observed in African rice ([21], De Waele, personal communication). Sexual compatibility and hybrid sterility limit the effort of combining useful traits from these two rice species. Fertility of the hybrids can be restored by repeated backcrossing, but there is a risk of losing the desirable traits [28].

Nevertheless, recently, crosses and host-response evaluation experiments at the International Rice Research Institute (IRRI, Los Baños, Philippines) resulted in the identification of some promising Asian rice genotypes derived from *O. sativa* parents that are either resistant to and/or tolerant of *M. graminicola.* Resistance to *M. graminicola* in rice has earlier been reported to be quantitative in nature or governed by many genes with additive effects. Shrestha et al. [29] reported QTLs associated with root galling on five (1, 2, 6, 7 and 9) chromosomes using RILs derived from a cross of Bala and Azucena, both *Oryza sativa* accessions. Variance explained by significant QTLs ranged from 8.3 to 10.3%. Another QTLs associated with the number of root galls per root system, eggs per root system and eggs per gram of roots on chromosomes 1 and 3 were reported by Jena et al. [30] using RILs derived from a cross of Annapurna and Ramakrishna, both traditional rice from India, whereas recently, QTLs also associated with root galling were mapped on chromosomes 1, 3, 4, 5, 11 and 12 by Dimpka et al. [27] from a diverse rice panel. The first nematode resistance gene reported in rice was *Hsa-1*^*Og*^, which confers resistance to the cyst nematode, *Heterodera sacchari*. This gene is located on chromosome 11 and was identified from a segregating population derived from TOG5681 and IR64 [31]. TOG5681 is an *O. glaberrima* accession that is resistant to *M. graminicola* infection [32].

Following the terminology of Bos and Parlevliet (1995), resistance/susceptibility and tolerance/sensitivity are defined as independent, relative qualities of a host plant based on comparison between genotypes. A host plant may either suppress/limit (resistance) or allow (susceptibility) nematode development and reproduction; it may suffer either little injury (tolerance), even when heavily infected with nematodes, or much injury (sensitivity), even when relatively lightly infected with nematodes. Resistance/susceptibility can be determined by measuring nematode reproduction on, and especially, in the roots, whereas tolerance/sensitivity can be determined by measuring the effect of nematode population on plant growth and yield-contributing traits and/or on yield [33]. Breeding for both resistance and tolerance will facilitate development of rice cultivars that will not only suppress/limit nematode reproduction but that will also incur acceptable yield reduction (< 10%) despite the presence of *M. graminicola* infection.

This study was undertaken to identify and map QTLs that confer resistance to or tolerance for *M. graminicola*. Important parameters for nematode resistance such as the nematode reproduction in the roots (J2 per root system and J2 per g of root) and nematode tolerance measured by yield reduction were studied. QTL for nematode resistance and tolerance using these parameters were not previously reported. We aimed to explore the individual loci affecting nematode resistance or tolerance, root galling and some important plant growth and yield-contributing plant traits. These QTLs are necessary in designing a marker-assisted breeding (MAB) program for resistance to and tolerance for *M. graminicola*. Such a program is expected to accelerate the development and deployment of cultivars with resistance to and tolerance for *M. graminicola.*

## Methods

### Plant materials and population development

The RILs were developed from a cross involving IR78877–208-B-1-2 as male parent and IR64 as female parent. IR78877–208-B-1-2 derived from a cross between Apo and IR72. Apo is a landrace from the Philippines and IR72 is a cultivar developed by IRRI. The resistance and tolerance in IR78877–208-B-1-2 were observed under both controlled (growth chamber) and field-simulated (outdoor concrete raised beds) conditions. IR64 is a cultivar also developed at IRRI, and is widely adapted and grown in South and Southeast Asia because of its high-yielding ability. However, IR64 is susceptible and sensitive to *M. graminicola*, showing high yield losses in nematode-infested fields.

One hundred twenty five F_1_ seeds from the cross were selfed to produce F_2_ seeds. One panicle per plant was harvested for all plants, then, 4 to 5 seeds from each panicle were taken and bulked and advanced to F_3._ Three hundred F_4_ plants were harvested, from which 20 seeds from each plant were taken and used in this experiment.

### Evaluation of the host response of the RIL population

Three hundred F_4_ RILs derived from IR78877–208-B-1-2 and IR64 were evaluated, along with the parents, for their host response to *M. graminicola* infection in nematode-infested and non-infested outdoor concrete raised beds (7 m long, 1.1 m wide and 0.15 m deep) in Los Baños during the dry seasons of 2012 and 2013. The *O. glaberrima* genotype TOG5674 was included as the resistant reference genotype, whereas the *O. sativa* genotype UPLRi-5 was included as the susceptible reference genotype. Previous studies showed that TOG5674 was highly resistant and UPLRi-5 was highly susceptible to *M. graminicola* across different experimental conditions ([20], De Waele personal communication). Fourteen concrete beds were used. Each bed was filled with 1350 kg of heat-sterilized soil (a 1:1 mixture of garden soil and sand). Seven beds were infested with *M. graminicola* and seven remained non-infested. For infestation of the soil in the beds, 175 g of finely chopped roots of UPLRi-5 infected with *M. graminicola* were evenly distributed on top of a levelled 10-cm-thick soil layer, and then covered with a thin layer of soil. The initial inoculum (Pi) in each infested bed was equivalent to 1 s stage juvenile (J2) per g of soil. J2 is the infective stage of *M. graminicola*. The *M. graminicola* population was originally isolated from a rice plant (name unknown) growing in an infested rice field in Batangas, Philippines. The population was established from a single egg mass and maintained on UPLRi-5 in soil pots in one of IRRI’s greenhouses. The seeds of the 300 F_4_ RILs were separately germinated in petri-dishes at a room condition. Five-day-old pre-germinated seeds of the 300 F_4_ RILs and their two parents as well as the resistant reference TOG5674 and susceptible reference UPLRi-5 were planted in rows arranged in an alpha lattice design with two replications. Each bed was divided into 2 columns of rows, thus each bed had 88 rows. There were 3 hills spaced at 15 cm in each row. Rows were spaced at 15 cm. Each hill was planted with two pre-germinated seeds, which were thinned to one, 1 week after planting. Fertilizer was applied at 14, 35 and 55 days after planting (DAP) at a rate of 120–60-60 NPK kg/ha. Aerobic condition was maintained in all beds throughout the experiment. In an aerobic condition, rice plants are grown in a well-drained, non-puddled and non-saturated soil. Irrigation was applied to bring the soil water content in the root zone up to field capacity. A rat fence, rat baits and bird nets were also installed to protect the plants from rat and bird damage.

At harvest (approximately 12 weeks after planting for TOG5674 and 15 weeks after planting for other genotypes), the root system of each plant was carefully removed from the soil and washed with tap water to remove all adhering soil particles. The severity of root galling was recorded using a 0–5 scale where 0 = no galls; 1 = < 10% of the root system galled and 2 = 10–25%, 3 = 26–50%, 4 = 51–75% and 5= > 50% of the root system galled [34]. After the fresh root weight of each plant was recorded, the roots were cut into about 0.5-cm pieces and placed in a mistifier chamber at an ambient temperature of 27 °C. [36]. The extracted nematodes were collected at 7 and 14 days after the roots were placed in the mistifier. A sub-sample of 1 ml was used for counting the number of J2 extracted from each root system. An average of two counts was used to determine the final nematode population density (Pf). The Pf divided by the fresh root weight of each root system gave the number of J2 per root unit (1 g of fresh roots). The average number of J2 per root system and J2 per g of roots of each plant was compared with the average number of J2 per root system and J2 per g of roots of the resistant and susceptible reference genotypes to determine their host response. Classification of the host response of the RILs as resistant, partially resistant, susceptible or inconclusive was based on the methodology used by Dochez et al. [35] (Table 1).

**Table 1:** Classification of the host response of RILs to *Meloidogyne graminicola* infection based on a comparison with the host response of the susceptible reference UPLRi-5^S^ and the resistant reference TOG5674^R^

Statistical difference with UPLRi-5^S^	Statistical difference with TOG5674^R^	Host response
Significant(*)	Not significant	Resistant (R)
Significant	Significant	Partially resistant (PR)
Not significant (ns)	Significant	Susceptible (S)
Not significant	Not significant	Inconclusive (I)

*Significant according to LSD (*P* ≤ 0.05)

Plant growth and yield-contributing traits (fresh and dry shoot weight, number of panicles per plant, percentage of filled grains per panicle and grain yield per plant) were measured. Yield reduction (YR) was determined to assess the level of tolerance for *M. graminicola* infection of each genotype using the following scale: < 10% YR = tolerant; 10–20% YR = less sensitive; 21–30% YR = sensitive and > 30% YR = highly sensitive. Yield reduction was computed as:

$$ \left[\begin{array}{l}\left( yield\ of\ plants\ grown\ in\ non- infested\ soil- yield\ of\ plants\ grown\ in\ in fested\right)/\\ {} yield\ of\ plants\ grown\ in\ non- infested\ soil\end{array}\right]\times 100. $$

Grain yields are reported at 14% moisture content.

### Genotyping of the RIL population

Genomic DNA was bulked from the leaf samples collected from all the plant replicates of each genotype at 14 DAP. DNA extraction was carried out following the modified CTAB method [37]. The quantity and quality of the DNA samples was checked in 1% agarose gel. DNA samples were sent to LGC Genomics (UK) for SNP genotyping. There were 1998 SNPs used for the polymorphism survey between the parents IR78877–208-B-1-2 and IR64. Of these, 600 (30%) were polymorphic but only 134 SNPs were selected to genotype the whole population. Selection of the markers was based on their distribution throughout the chromosomes. In addition to SNP markers, 35 polymorphic SSR markers were added to saturate some chromosomal regions with no available SNPs. All 169 molecular markers were distributed among 12 rice chromosomes covering 2027 cM with an average intermarker distance of 11.9 cM.

### Statistical analysis of the phenotypic data

Genotype means were estimated from each trial (season x treatment) using the following mixed model.

$$ {y}_{ijk}=\mu +{g}_i+{r}_j+{b}_{lj}+{e}_{ijk}; $$

where *y*_*ijk*_ is the performance of the i^th^ genotype in the k^th^ block of the j^th^ replication; *μ* represents the overall mean; *a*_*i*_ represents the effect of *i*^*th*^ genotype; *r*_*j*_ represents the effect of *j*^*th*^ replicate; *b*_*lj*_ the effect of *l*^*th*^ block within the *j*^*th*^ replicate; and *e*_ijk_ represents the random error. The distribution of the random effects is as follows:

$$ {g}_i\sim N\left(0,{\sigma^2}_g.I\right);{r}_j\sim N\left(0,{\sigma^2}_rI\right);{b}_{jk}\sim N\left(0,{\sigma^2}_bI\right);{e}_{ijk}\sim N\left(0,{\sigma^2}_eI\right) $$

The variance-covariance structure of *y* vector is given by:

$$ V(y)={\sigma^2}_g.I+{\sigma^2}_r.I+{\sigma^2}_b.I+{\sigma^2}_e.I $$

*σ*^2^_*g*_ is the genotypic variance, *σ*^2^_*r*_ is the variance of the replicates, *σ*^2^_*b*_ is the variance of the blocks within replicates, *σ*^2^*e* is the error variance and *I* indicates the identity matrix. The model was fitted using the PBtools. Normality and homogeneity of variance of the response variable was checked using diagnostic residual plots. Data was transformed when the residuals from the fitted model did not meet the assumptions. This is indicated by (i) a non-random scatter of points around the ‘0’ line on the residuals versus fits plot on which the residuals appear on the y axis and the fitted values appear on the x axis and (ii) the residuals that did not fall roughly on a straight line on a QQ plot. Correlation coefficients among traits were calculated by Pearson analysis using SPSS v16.0 (SPSS Inc., 2007).

### QTL analysis

The number of J2 per root system, J2 per g of roots and the severity of root galling were used to map QTLs for resistance, whereas percentage of yield reduction was used to map QTLs for tolerance. Plant growth and yield-contributing traits such as fresh and dry root and shoot weight, number of panicles per plant, percentage of filled grains per plant and yield per plant of the infected plants were also included in the QTL analysis. Broad-sense heritability (H) of each trait was computed using:

$$ H=\frac{\sigma^2g}{\sigma^2g+\left(\frac{\sigma^2e}{r}\right)} $$

Where: *σ*^2^*g* = genetic variance, *σ*^2^*e* = residual variance and *r* = number of replications.

A linkage map was constructed using ICiMapping v3.1 software [38] with polymorphic SNP and SSR markers between the parents IR78877–208-B-1-2 and IR64. Linkage groups were identified using the Group command to identify linkage groups with a logarithm of odds (LOD) score of 3.0, and recombination frequency was converted into centimorgans using the Kosambi mapping function. QTLs responsible for resistance and tolerance to *M. graminicola* were analyzed with the Multiple trait Multiple Interval Mapping (MT-MIM) using the QGENE 4.3.10 software [39]. Pleiotropy effect was likewise analysed using QGENE. MT-MIM analysis was similar to that of multiple-trait composite interval mapping (MT-CIM) analysis, however, in MT-CIM, only one QTL is tested at a time whereas MT-MIM tests more than one QTL (denoted as ‘q’ in the formula) at a time. The model for MT-MIM was:

$$ {\displaystyle \begin{array}{c}Y\\ {}n\;x\;t\end{array}}=\sum {\displaystyle \begin{array}{c}q\\ {}i=1\end{array}}\left(\begin{array}{c}{x}_i\\ {} nx1\end{array}\kern0.48em \begin{array}{c}{a}_i\\ {}1 xt\end{array}+\begin{array}{c}{z}_i\\ {} nx1\end{array}\right)+{\displaystyle \begin{array}{c}E\\ {}n\;x\;t\end{array}} $$

where ‘q’ is the number of QTLs being fitted simultaneously, t is the number of analyzed traits, ‘n’ is number of observation, ‘ai’ is the additive effects of a QTL, and ‘E’ is random error. Co-locating QTLs were tested in pairs in the same QTL region to test for pleiotropy effects. LOD threshold at 5 and 1% for pleiotropy effect was determined using 1000 permutations. Pleiotropy effect is assumed when LOD threshold is significant. Epistasis was analyzed using QTL Network 2.0 [40]. The first and second dimensional genome scan function was used to map for epistatic interactions. The parameters used in the analysis were 1000 permutations, experimental-wise significance level of 0.05 for detection of QTLs with their effect, genome scan configuration (1.0 cM walk speed,10.0 cM testing window and filtration window size) and Monte Carlo Markov Chain (MCMC) for estimating QTL effects. Mapped QTLs were named after McCouch et al. [40].

## Results

### Host response of the RIL population

**Fig. 1:**
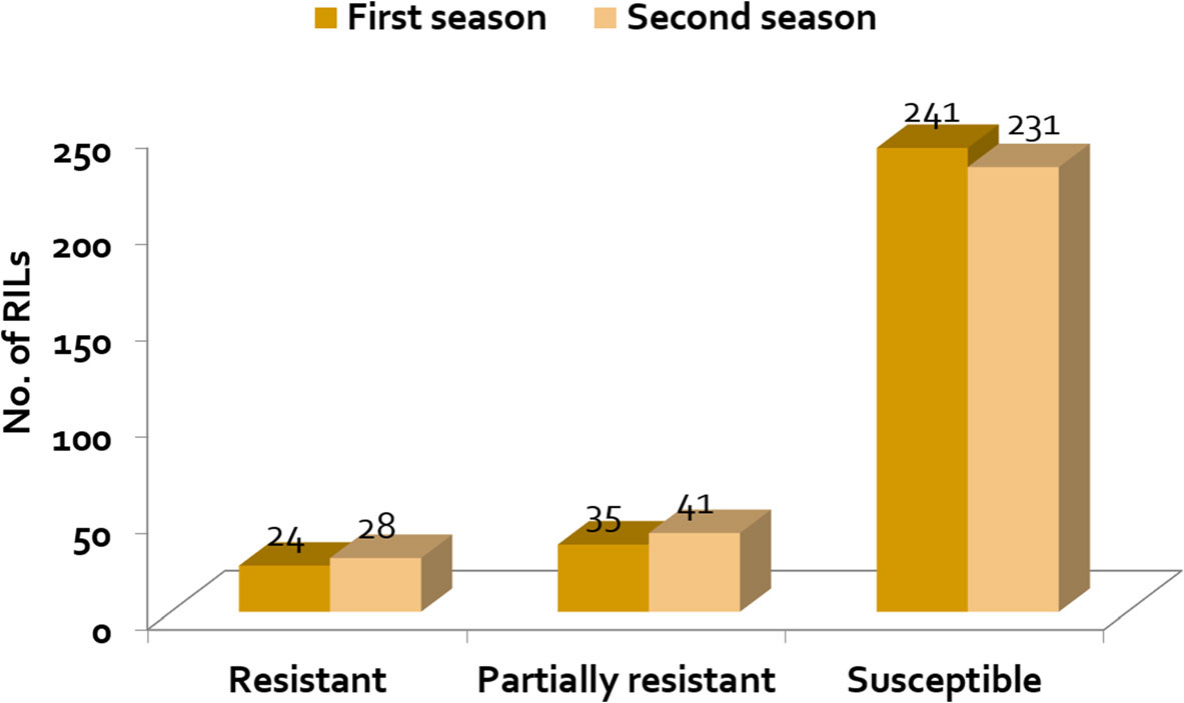
Host response in terms of resistance of the 300 RILs evaluated in *M. graminicola* inoculated concrete beds at IRRI

A high variation in host response to *M. graminicola* infection was observed among the RILs during our two-season study. In the 1st season, on the basis of J2 per root system, 24 (8%) RILs were identified as resistant, 35 (12%) as partially resistant and 241 (80%) as susceptible (Fig. 1). J2 per root system averaged 802 J2 in the resistant RILs, 3629 J2 in the partially resistant and 21,376 J2 in susceptible RILs. Root gall rating averaged 3.4, 4.3 and 4.6 in the resistant, partially resistant and susceptible RILs, respectively. Nematode reproduction was higher in IR64 (54,368 J2 per root system), than in IR78877–208-B-1-2 (4688 J2 per root system). TOG5674 had the lowest nematode reproduction (125 J2 per root system). On the basis of the number of J2 per g of roots, 28 (9%) of the RILs examined were resistant, 34 (12%) were partially resistant and 238 (79%) were susceptible. J2 per g roots averaged 83, 303 and 1687 in the resistant, partially resistant and susceptible RILs, respectively (Table 2).

**Table 2:** Average root weight, severity of root galling and nematode (J2) reproduction in parental lines and RILs infected with *Meloidogyne graminicola* as assessed in two seasons

Traits	First season				Second season			
	Root wt.	Gall rating	J2 per root system	J2 per g of root	Root wt.	Gall rating	J2 per root system	J2 per g of root
IR64	11.8	5.0	54,368	4620	16.1	5.0	44,012	4113
IR78877–208-B-1-2	15.6	3.0	4688	372	13.8	3.0	3263	359
F_4_ RILs								
Resistant	12.8	3.4	802	66	15.4	3.5	1696	114
Partially resistant	12.9	4.3	3629	309	15.3	4.1	5789	400
Susceptible	15.6	4.6	21,373	1544	15.9	4.4	20,207	1358
H (%)	69	68	89	88	65	64	94	84

In the 2nd season, 28 (9%) of the RILs were resistant, 41 (14%) partially resistant and 231 (77%) susceptible. J2 per root system averaged 1696 in the resistant RILs, 5789 in the partially resistant and 20,207 in the susceptible RILs. Root gall rating averaged 3.5 in the resistant RILs, 4.1 in the partially resistant and 4.4 in the susceptible RILs. Based on J2 per g of roots, 26 (8%) RILs were resistant, 41 (14%) were partially resistant and 233 (78%) were susceptible. J2 per g of roots averaged 135, 375 and 1585 in the resistant, partially resistant and susceptible RILs, respectively. Again, J2 per root system and J2 per g of roots was higher in IR64 (44,012 and 4113, respectively) than in IR78877–208-B-1-2 (3263 and 359, respectively). Estimated heritability of root weight, root gall rating and nematode reproduction per root system and per g roots were relatively high in both seasons (69, 68, 89 and 88, respectively, in the 1st season and 65, 64, 94 and 84, respectively, in the 2nd season).

There were 18 RILs that were found consistently resistant out of the 24 and 28 resistant in the first and second season respectively while 14 RILs were consistently partially resistant out of the 35 and 41 partially resistant in the first and second season respectively. Most of those not consistent became susceptible during the second season study. This means that nematode reproduction in the same rice genotype may vary despite the same experimental set-up. The reasons for these variations remain unknown. They may be caused by differences in “vitality” of the nematode inoculum, like for instance, the same *M. graminicola* culture (population) may produce a different number of offspring at different times of the culturing. Other sources of inoculum such as *M. graminicola* eggs and egg-laying females may also vary in root inoculum from one to another experiment.

**Fig. 2:**
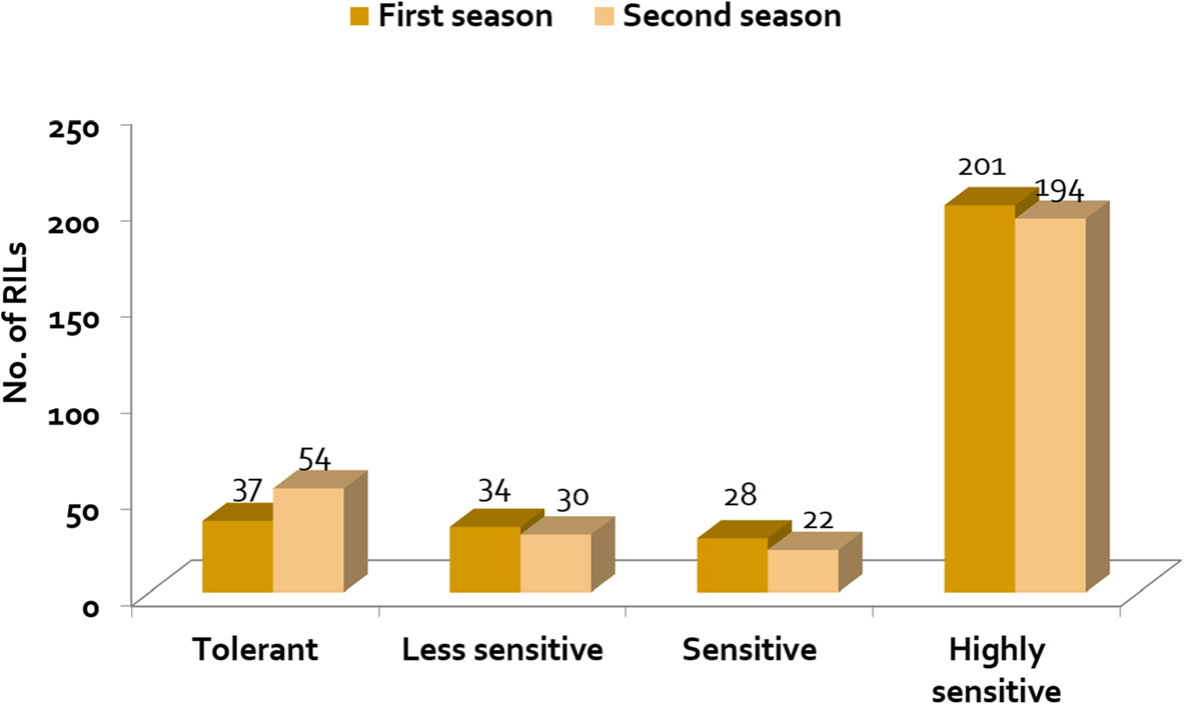
Host response in terms of tolerance of the 300 RILs evaluated in *M. graminicola* inoculated concrete beds at IRRI

Based on percentage of yield reduction in the 1st season, 37 RILs were identified as tolerant (˂ 10% YR), 34 were less sensitive, 28 were sensitive and 201 were highly sensitive. In the 2nd season, 54 RILs were tolerant, 30 were less sensitive, 22 were sensitive and 194 were highly sensitive (Fig. 2). The average percentage of yield reduction in the 1st season was 6, 16, 26 and 52% for tolerant, less sensitive, sensitive, and highly sensitive RILs, respectively; in the 2nd season, these values were 5, 17, 29 and 49%, respectively. IR64 showed a high yield reduction in both seasons (66 and 46%) and was categorized as highly sensitive. In contrast, IR78877–208-B-1-2 was consistently tolerant, showing a yield reduction of only 3 and 2% in the 1st and 2nd seasons, respectively. T-test analysis revealed that, in both seasons, the average number of panicles per plant and percentage of filled grains per plant of the infected plants were significantly (*P* ≤ 0.05) reduced in the less sensitive, sensitive and highly sensitive RILs, but not in the tolerant RILs, which indicates that these yield-contributing traits were not affected by nematode inoculation in tolerant RILs (Table 3).

**Table 3:** Average yield and yield reduction of *M. graminicola* inoculated (I) and un-inoculated (UI) parental lines and RILs in two seasons

Traits	First season			Second season		
	Plant yield (g)		YR (%)	Plant yield (g)		YR (%)
	UI	I		UI	I	
IR64	15.5	5.2	66.4^*^	16.3	8.8	46.0^*^
IR78877-208-B-1-2	14.1	13.7	2.8 ns	12.6	12.3	2.4 ns
F_4_ RILs						
Tolerant	15.8	14.8	6.3 ns	18.0	17.1	5.3 ns
Less sensitive	14.8	12.4	16.0 ns	16.9	14.7	17 ns
Sensitive	16.1	12.0	25.7^*^	17.0	12.2	28.6^*^
Highly sensitive	18.8	9.1	52.0^*^	20.6	10.3	49.2^*^

*UI* Un-inoculated, *I* Inoculated, *YR* Yield reduction

^*^indicates that the % reduction is significant according to LSD (*P* ≤ 0.05)

**Fig. 3:**
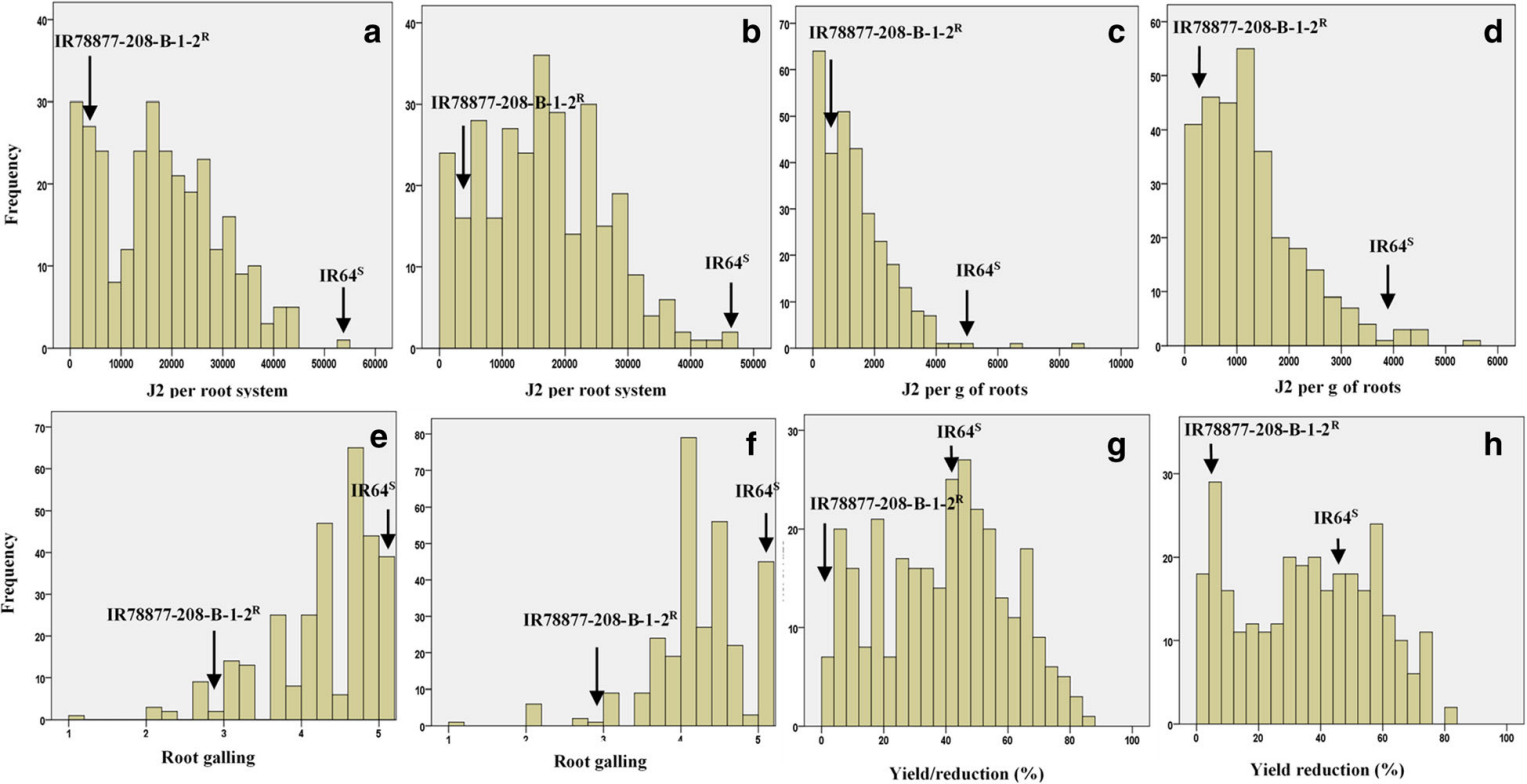
Frequency distribution forJ2 per root system in the first (**a**) and second (**b**) seasons, J2 g of roots in the first (**c**) and second (**d**) seasons, root galling in the first (**e**) and second (**f**) seasons, and yield reduction (%) in the first (**g**) and second (**h**) seasons

The phenotypic distributions of the plant traits examined showed a wide range of variation and transgressive segregation, indicating the presence of polygenic resistance. In both seasons, transgressive segregants were present, showing better resistance and tolerance or had lower nematode reproduction, lower root galling severity and lower percentage of yield reduction compared with IR78877–208-B-1-2. A few transgressive segregants showing a higher susceptibility (based on J2 per g of roots) and a higher percentage of yield reduction compared with IR64 were also observed (Fig. 3).

### Phenotypic correlations

J2 per root system and per g of roots were negatively (*P* ≤ 0.01) correlated with dry shoot weight, and fresh and dry root weight in both seasons, whereas root gall rating showed a negative correlation (*P* ≤ 0.05) with fresh and dry shoot weight, number of panicles per plant, yield per plant and percentage of yield reduction in both seasons (Table 4).

**Table 4:** Correlation of nematode reproduction and gall rating with agronomic traits for the 1st and 2nd seasons

	First season			Second season		
	J2 per root system	J2 per g of root	Gall rating	J2 per root system	J2 per g of root	Gall rating
Plant height	−0.053	− 0.198^**^	− 0.072	−0.021	− 0.039	−0.145^*^
Fresh shoot wt.	− 0.048	− 0.288^**^	− 0.262^**^	− 0.023	−0.068	− 0.175^**^
Dry shoot wt.	− 0.154^**^	− 0.263^**^	− 0.286^**^	− 0.246^**^	− 0.256^**^	− 0.189^**^
Fresh root wt.	− 0.177^**^	− 0.341^**^	0.021	−0.180^**^	− 0.428^**^	0.006
Dry root wt.	−0.155^**^	− 0.347^**^	− 0.051	−0.158^**^	− 0.389^**^	− 0.023
Panicles per plant	−0.034	− 0.188^**^	− 0.140^*^	− 0.010	−0.046	− 0.114^*^
percentage of filled grains	−0.053	− 0.083	−0.067	− 0.059	−0.023	− 0.067
Yield per plant	−0.005	− 0.057	−0.172^**^	− 0.012	−0.082	− 0.151^**^
Yield reduction	0.046	0.022	0.173^*^	0.013	0.077	0.245^**^

**significant at *P* = 0.01

*significant at *P* = 0.05

### QTL analysis

**Fig. 4:**
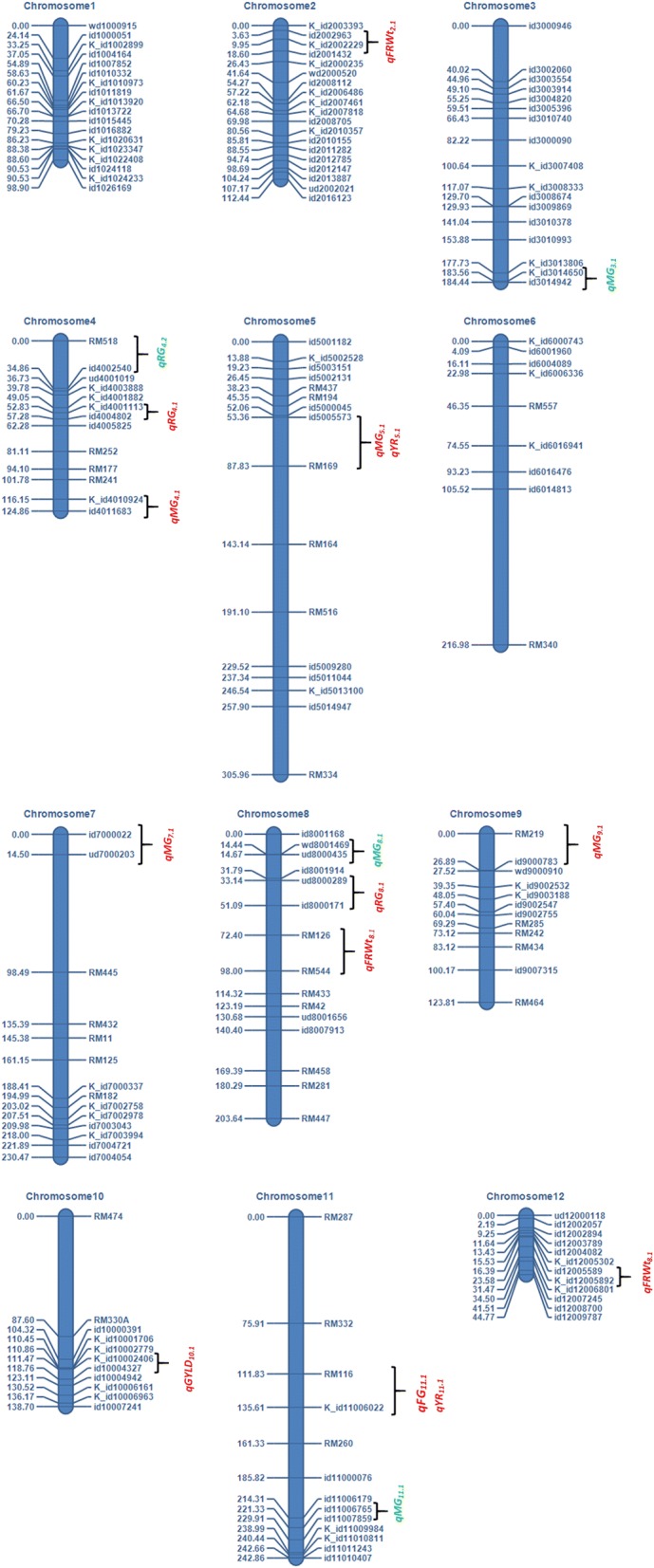
QTLs for resistance (*qMGR, qRG*), tolerance (*qYR*) and agronomic traits detected from RILs derived from IR64/IR78877–208-B-1-2. Red fonts are QTLs with main effects and green fonts are involved in epistatic interaction

**Fig. 5:**
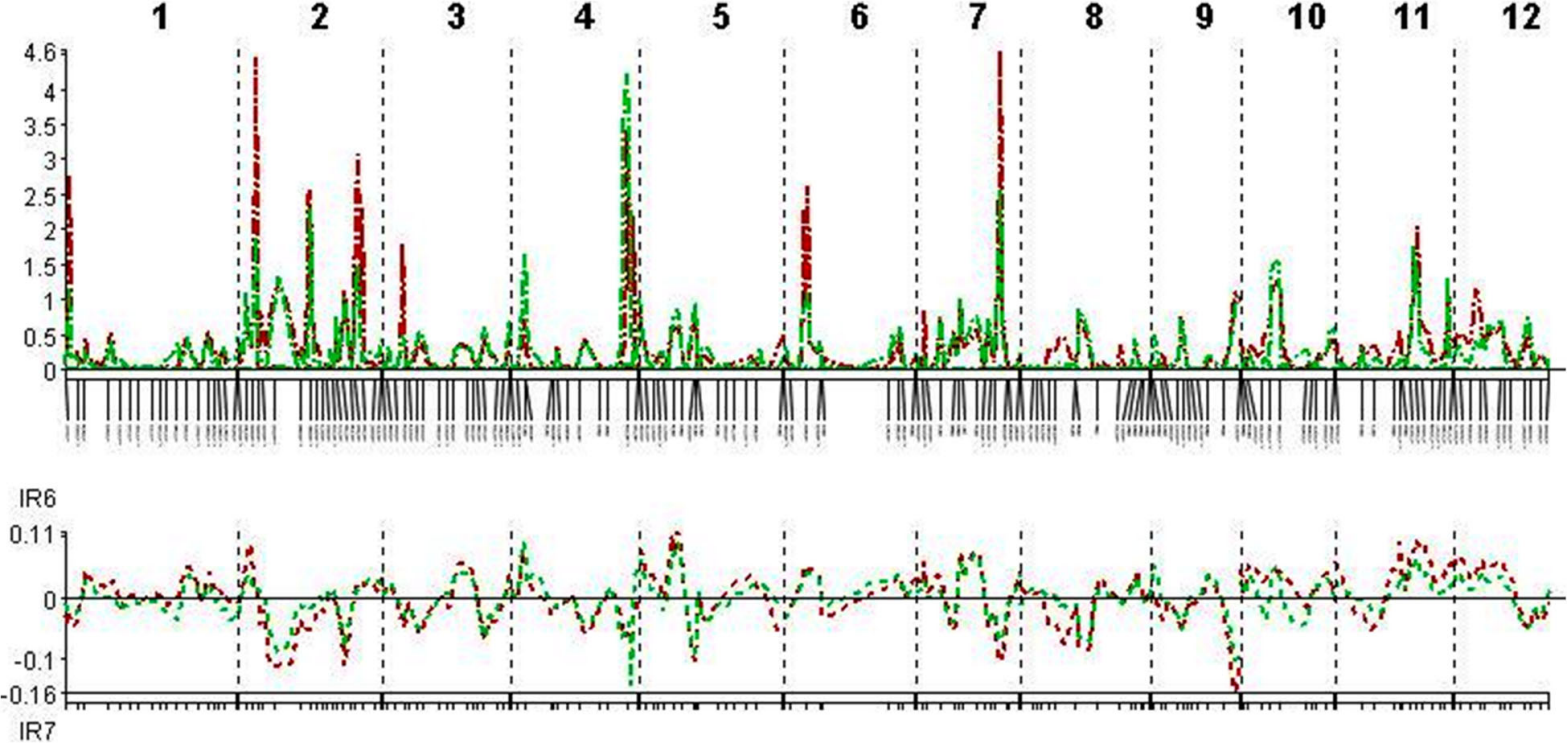
QTL likelihood curves of LOD scores for J2 per root system for the first (red line) and second (green line) season

**Fig. 6:**
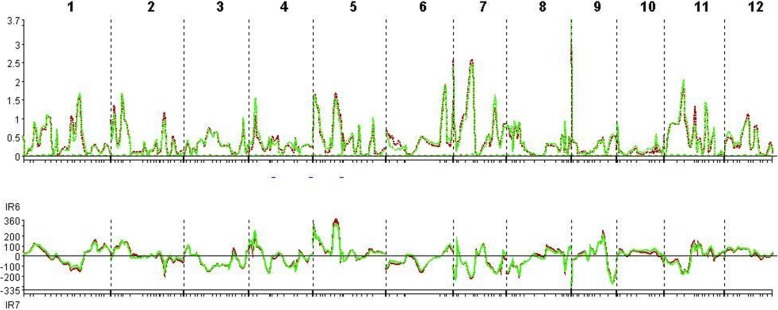
QTL likelihood curves of LOD scores for J2 per g of roots for the first (red line) and second (green line) season

**Fig. 7:**
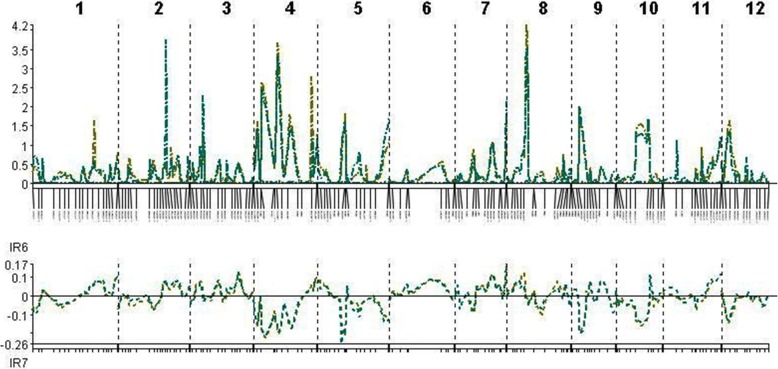
QTL likelihood curves of LOD scores for root galling for the first (red line) and second (green line) season

**Fig. 8:**
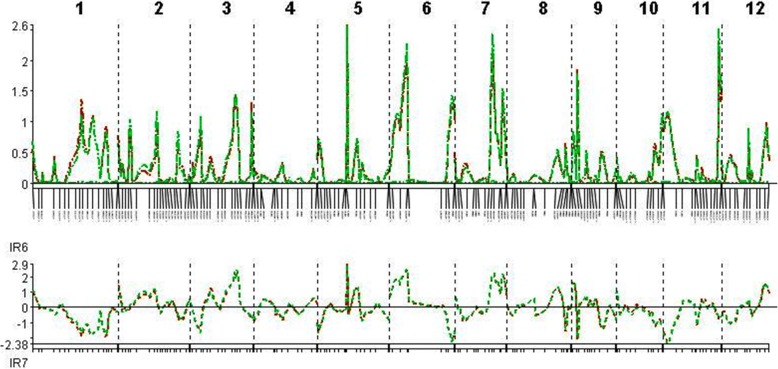
QTL likelihood curves of LOD scores for yield reduction (%) for the first (red line) and second (green line) season

**Fig. 9:**
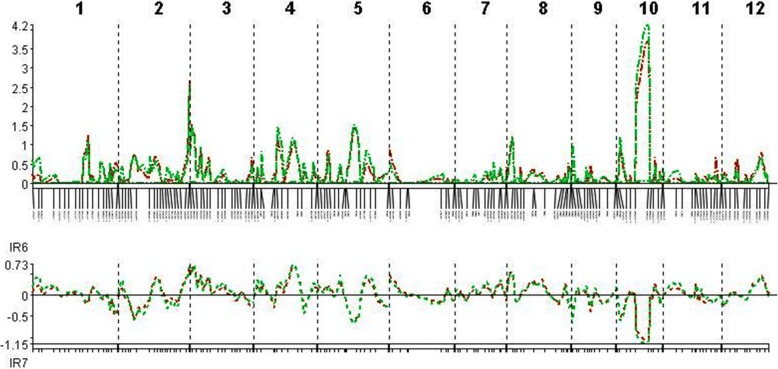
QTL likelihood curves of LOD scores for grain yield for the first (red line) and second (green line) season

**Fig. 10:**
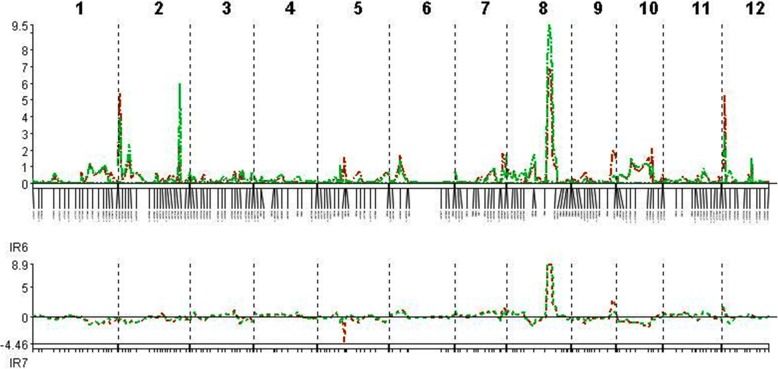
QTL likelihood curves of LOD scores for fresh root weight (%) for the first (red line) and second (green line) season

The QTLs associated with resistance to and tolerance for *M. graminicola*, and plant growth and yield-contributing traits are summarized in Table 5 and Fig. 4. LOd curves of these QTLs in two seasons are also presented (Figs. 5, 6, 7, 8, 9, 10). In the 1st season, based on the number of J2 per root system, QTLs were mapped on chromosomes 4 (*qMGR*_*4.1*_), 7 (*qMGR*_*7.1*_) and 9 (*qMGR*_*9.1*_) in intervals of K_id4010924 – id4011683, K_id7002978 - id7003043, and RM219 – id9000783. These loci explained 5.1, 6.8 and 4.8 of the phenotypic variance respectively. QTLs affecting severity of root galling were found on chromosomes 4 (*qRG*_*4.1*_) and 8 (*qRG*_*8.1*_). *qRG*_*4.1*_ was flanked by K_id4001113 – id4004802, explaining 7% of the variance, whereas *qRG*_*8.1*_ was flanked by ud8000289 – id8000171, explaining 6.2% of the variance. Two QTLs that responsible for the percentage of yield reduction were located on chromosomes 5 (*qYR*_*5.1*_) and 11 (*qYR*_*11.1*_) in intervals of id5005573 - RM 169 and RM116 – K_id11006022 and which accounted for 4.1 and 3.5% of the phenotypic variance respectively. A QTL affecting grain yield (*qGYLD*_*10.1*_) was found in intervals of K_id10002406 - id10004327 and explained 5.5% of the variance while a QTL affecting filled grains (*qFG*_*11.1*_) was found in intervals of id5005573 - RM 169 on chromosome 11 and explained 5.5% of the variance. Three QTLs were found associated with fresh root weight on chromosomes 2 (*qFRWt*_*2.1*_), 8 (*qFRWt*_*8.1*_), and and 12 (*qFRWt*_*12.1*_) in intervals of id2002963 – k_id2002229, RM126 – RM544 and id12005589 – k_id12005892 and accounted for 8.0, 10.0 and 7.7% of the phenotypic variance respectively.

**Table 5:** QTLs associated with agronomic traits and root-knot nematode, *M. graminicola*, resistance and tolerance in two seasons

Trait	Chromosome	QTL name	Interval Marker	Peak marker	Position	Add effect	LOD	R^2^ (%)	Donor of allele
1st season									
J2RS	4	*qMGR*_*4.1*_	K_id4010924 – id4011683	id4011683	121.7	− 3850.0	3.4	5.1	IR78877–208-B-1-2
	7	*qMGR*_*7.1*_	K_id7002978 - id7003043	id7003043	2.3	− 4078.3	4.6	6.8	IR78877–208-B-1-2
J2GRT	9	*qMGR*_*9.1*_	RM219 – id9000783	RM219	0.0	− 365.2	3.2	4.8	IR78877–208-B-1-2
RG	4	*qRG*_*4.1*_	K_id4001113 – id4004802	id4004802	55.7	−0.3	4.7	7.0	IR78877–208-B-1-2
	8	*qRG*_*8.1*_	ud8000289 – id8000171	ud8000289	40.0	−0.2	4.1	6.2	IR78877–208-B-1-2
YR (%)	5	*qYR*_*5.1*_	id5005573 - RM 169	RM169	58.3	−0.7	2.6	4.1	IR78877–208-B-1-2
	11	*qYR*_*11.1*_	RM116 – K_id11006022	RM116	111.5	−0.7	2.3	3.5	IR78877–208-B-1-2
GYLD	10	*qGYLD*_*10.1*_	K_id10002406 - id10004327	id10004327	119.0	1.1	3.7	5.5	IR64
FG (%)	11	*qFG*_*11.1*_	id5005573 - RM 169	RM169	123.5	3.4	3.7	5.5	IR64
FRWT	2	*qFRWt*_*2.1*_	id2002963 – k_id2002229	id2002963	5.2	−0.25	5.4	8.0	IR78877–208-B-1-2
	8	*qFRWt*_*8.1*_	RM126 – RM544	RM544	86	8.8	6.9	10.0	IR64
	12	*qFRWt*_*12.1*_	id12005589 – k_id12005892	id12005589	17.5	1.24	5.2	7.7	IR64
2nd season									
J2RS	4	*qMGR*_*4.1*_	K_id4010924 – id4011683	id4011683	121.7	− 4050.0	4.2	6.3	IR78877–208-B-1-2
	7	*qMGR*_*7.1*_	K_id7002978 - id7003043	id7003043	82.3	− 2070.3	2.6	4.0	IR78877–208-B-1-2
J2GRT	9	*qMGR*_*9.1*_	RM219 – id9000783	RM219	0.0	−341.2	3.7	5.5	IR78877–208-B-1-2
RG	4	*qRG*_*4.1*_	K_id4001113 – id4004802	id4004802	35.7	−0.2	4.5	6.6	IR78877–208-B-1-2
	8	*qRG*_*8.1*_	ud8000289 – id8000171	ud8000289	40.0	−0.2	3.5	5.2	IR78877–208-B-1-2
YR (%)	5	*qYR*_*5.1*_	id5005573 - RM 169	RM169	58.3	−0.8	2.6	4.2	IR78877–208-B-1-2
	11	*qYR*_*11.1*_	RM116 – K_id11006022	RM116	111.5	−0.7	2.5	3.8	IR78877–208-B-1-2
GYLD	10	*qGYLD*_*10.1*_	K_id10002406 - id10004327	id10004327	119.0	1.2	4.2	6.3	IR64
FG (%)	11	*qFG*_*11.1*_	id5005573 - RM 169	RM169	123.5	3.3	3.4	5.2	IR64
FRWT	2	*qFRWt*_*2.1*_	id2002963 – k_id2002229	id2002963	5.2	−0.36	3.9	5.9	IR78877–208-B-1-2
	8	*qFRWt*_*8.1*_	RM126 – RM544	RM544	86	8.5	9.5	14.0	IR64
	12	*qFRWt*_*12.1*_	id12005589 – k_id12005892	id12005589	17.5	1.24	3.0	4.5	IR64

*J2RS* J2 per root system, *J2GRT* J2 per g of root, *RG* root galling, *YR* yield reduction, *GYLD* yield per plant, *FG* filled grains, *FRWT* fresh root weight, *DRWT* dry root weight, *LOD* logarithm of odd (probability of linkage/probability of no linkage), *R*^*2*^ phenotypic variance explained by the QTL

In the 2nd season, the QTLs detected in the 1st season were confirmed.. Confirmed QTLs in the 2nd season were mapped by the same markers and had retained their positions observed in the 1st season. IR78877–208-B-1-2 has contributed the allele that affects resistance, severity of root galling, percentage of yield reduction on chromosome 5 and fresh root weight on chromosome 2. IR64 has contributed the alleles that affect percentage of yield reduction on chromosome 11, grain yield, percentage of filled grains and fresh root weight on chromosomes 8 and 12. It is interesting that some QTLs of different traits are located on the same chromosome. For example, a QTL affecting nematode reproduction (*qMGR*_*4.1*_) co-localized the QTL for root galling (*qRG*_*4.1*_). A QTL for tolerance (*qYR*_*11.1*_) also co-localized the QTL for filled grains on chromosome 11.

Co-locating QTLs such as the QTL for root galling and fresh root weight and QTL for tolerance and %filled grains were analyzed for pleiotropic effect, however, none from the LOD values of pleiotropic effects from the MT-MIM analysis was significant. Analysis on epistasis showed no epistatic interaction among and with the main QTLs for all traits. Two additive-by-additive interactions (*qMGR*_*3.1*_/ *qMGR*_*11.1*_ and *qMGR*_*4.2*_/*qMGR*_*8.*_1) involving 4 loci on chromosomes 3, 4, 7 and 11 were detected affecting resistance to *M. graminicola*. These interactions that involved IR78877–208-B-1-2 alleles were accountable for 2.5 and 3.1% of the phenotypic variance in the first season and 2.1 and 3.4% in the second season respectively (Table 6).

**Table 6:** Epistasis detected for resistance to rice root-knot nematode, *M. graminicola* in two seasons

QTL_i^a^	Interval_i^b^	Position_i^c^	Range_i^d^	QTL_j^a^	Interval_i^b^	Position_j^c^	Range_j^d^	AA^e^	*P* value	R^2^ (%)
First season										
*qMGR*_*3.1*_	K_id3014650-id3014942	125	122.2–125.2	*qMGR*_*11.1*_	id11006765-id11007859	82.6	78.0–85.0	− 1552	0.000000	2.5
*qMGR*_*4.2*_	RM516-id4002540	15.7	13.8–15.7	*qMGR*_*8.1*_	Ud8000435-ud8001469	28.1	25.3–35.1	−286	0.000009	3.1
Second season										
*qMGR*_*3.1*_	K_id3014650-id3014942	125	121.2–125.2	*qMGR*_*11.1*_	id11006765-id11007859	82.6	77.0–86.0	−1552	0.000000	2.1
*qMGR*_*4.2*_	RM516-id4002540	15.7	13.8–15.7	*qMGR*_*8.1*_	Ud8000435-ud8001469	28.1	25.3–35.6	−286	0.000002	3.4

R^2^ is the phenotypic variance explained by the interaction

^a^QTL_i and QTL_j are the two QTL involved in interaction

^b^Interval_i and interval_j are the flanking markers of QTL_i and QTL_j respectively

^c^Position_i and position_j is the distance between QTL_i/QTL_j and the first marker of the relevant chromosome

^d^Range_i and range_j is the position support interval of QTL_i and QTL_j respectively

^e^AA is the estimated additive by additive effect

## Discussion

### Host response of the RIL population

Host responses in terms of resistance to and tolerance for *M. graminicola* varied among the RILs. In our two-season study, we were able to identify RILs that are consistently resistant or partially resistant, and RILs that are consistently tolerant. A few RILs showed combined resistance and tolerance in both seasons. Some of the resistant RILs were not tolerant and vice versa. Of the 24 RILs that are resistant and 37 that are tolerant in the first season, there were 6 RILs that were both resistant and tolerant. Of the 28 RILs that are resistant and 54 that were tolerant in the second season, 5 RILs were both resistant and tolerant. Combining the two season study, only 4 RILs were consistently resistant and tolerant. This observation indicates an independent inheritance of resistance and tolerance in our population. As a result, resistant RILs were successful in limiting nematode reproduction but failed to achieve an acceptable yield because they were highly sensitive to nematode infection. Resistance to and tolerance for plant-parasitic nematodes might be simultaneously expressed but they can be inherited and expressed independently, resulting in plants that are resistant but sensitive, or tolerant but susceptible [33, 41]. This was demonstrated in other crops such as potato, in which resistance to and tolerance for the cyst nematodes *Globodera pallida* and *G. rostochiensis* were inherited independently [42–44]. Tolerance for *Heterodera glycines* identified in soybean also showed inheritance independent from resistance [45, 46]. The same was observed in resistance to and tolerance for *Rotylenchulus reniformis* [47] and *Meloidogyne incognita* in cotton [48].

### Phenotypic correlations

Correlation coefficient analysis showed that, except for percentage of yield reduction, nematode reproduction and severity of root galling were negatively correlated with root and shoot weight, number of panicles per plant, percentage of filled grains per plant and yield, suggesting that these traits were affected by nematode infection but to a different degree. Among these three variables, root galling correlated more with yield-contributing traits showing consistently negative significant (*P* ≤ 0.05) correlations with number of panicles per plant and yield per plant, as well as positive significant (*P* ≤ 0.05) correlations with percentage of yield reduction in both seasons.

On average, for the two seasons combined, percentage of reduction in plant height, fresh and dry shoot weight, and fresh and dry root weight of nematode-infected plants were 26, 41, 37, 18 and 24%, respectively. Significant reductions in these traits were also reported by Bimpong et al. [32].

### QTL analysis

Breeding for nematode resistance and tolerance is so far the safest and most economical option to alleviate plant damage and limit yield losses. With the advancement of molecular markers, numerous major genes and QTLs involved in nematode resistance and tolerance have been mapped from several crop species such as soybean, potato, tomato and pepper [49], but very limited information is available on QTLs for resistance to and tolerance for *M. graminicola* in rice. In our study, we report consistent QTLs on chromosomes 4, 5, 7 and 9 that increase nematode resistance by limiting the number of J2 population in the root. To our knowledge, there are no published reports using the number of J2 in the roots as a trait to map QTLs for resistance to *M. graminicola* on rice. QTLs related to severity of root galling on chromosomes 4 and 8 did not co-localize with the QTLs related to nematode reproduction, which suggest that in our RILs, population nematode reproduction and severity of root galling are controlled by different genetic loci. Co-locating QTLs such as the QTLs for root galling and fresh root weight and QTLs for tolerance and % filled grains were analyzed for pleiotropic effects, however, LOD obtained during the MT-MIM analysis was not significant suggesting there is no pleiotropic effect. No epistatic interactions were found among and with the main QTLs but there are two epistatic effects that involved pairs of loci that both lack main effects. The recombinant genotypes of both interactions tended to reduce the J2 reproduction in the root system. This suggests that while the main effects of each QTL appeared to serve as the major genetic basis in conferring resistance for both galling and J2 reproduction phenotypes, additive x additive epistatic interaction was important in suppressing nematode J2 reproduction. There has been no previous report on epistatic interaction associated with resistance and/or tolerance to the rice root-knot nematode, *M. graminicola*. In other crops, epistatic interaction was found responsible in suppressing egg production of *M. incognita* in cotton [61] and enhanced resistance to *Heterodera glycines* in soybean [62]. QTLs for severity of root galling were previously reported on chromosomes 1, 2, 6, 7, 9 and 11 [29] and chromosomes 1 and 3 [30]. In addition to this, QTLs associated to the number of eggs and eggs per g of roots that are very near the position of QTLs for severity of root galling were also identified [30]. All these QTLs were mapped from *indica* parents. The mapped QTLs related to the number of J2 per root system, J2 per g of roots and severity of root galling were not in the same location as the QTLs that have been previously reported, which indicate that the QTLs found in our population are new. In known *M. graminicola-*resistant genotypes, such as the African rice genotypes, resistance was associated with reduced J2 root penetration, delayed development of J2 that have penetrated the roots and lower reproduction of adult females [22]. In resistant Asian rice genotypes, retarded development of penetrating J2 and cell necrosis that further disrupts the development of the nematode’s feeding sites were the mechanisms of resistance [23]. In our study, low nematode reproduction in the roots, combined with lower severity of root galling, was observed in the resistant genotypes. Few RILs had better resistance than IR78877–208-B-1-2, the resistant parent. These transgressive segregants could have resulted from a recombination between the parents. Transgressive segregation was also observed in RILs by Shethra et al. [29] and Jena et al. [30]. Previous studies showed that some QTLs conferring resistance to rice diseases, such as the rice yellow mottle virus [56, 57], bacterial blight [49–51], blast [52, 53] and sheath blight [54, 55], and rice insects, such as the brown plant hopper [58], were also located on chromosomes 5 and 9. Interestingly, the QTL for rice blast and sheath blight on chromosome 9 is in almost the same location as *qMG*_*9.1*_ found in our study.

QTLs for tolerance of *M. graminicola* infection on chromosomes 5 and 11, based on percentage of yield reduction data, have not been reported before. QTLs that were found common in both seasons were derived from separate analysis and this may not necessarily infer stability of QTLs. Analysis on the variance across season would enhance the identification of QTLs which are specific to each season or which are stable across season. This will help the breeders to decide which QTL is more efficient to consider in a marker-assisted selection and breeding.

Yield reduction in nematode-infected rice plants can be attributed to a reduced number of productive tillers or panicles and a higher percentage of unfilled spikelets. In our study, number of panicles per plant and percentage of filled grains per plant have not been reduced in tolerant RILs, whereas these two traits were significantly reduced in sensitive and highly sensitive RILs. The formation of giant cells by J2, which eventually damaged the roots in the form of root galling, could have resulted in decreased panicles and percentage of filled grains. Damaged roots became inefficient to absorb and translocate water and other photosynthates that are crucial for the production of panicles and grain filling. Based on visual observation, heavily galled roots were shorter than those of the control plants. The hook-like terminal galls in the rice roots have prevented the roots to further elongate and this may have contributed to the inability of the plants to absorb and translocate water. Correlation coefficient analysis showed that severity of root galling is positively and significantly correlated with percentage of yield reduction, whereas it is negatively and significantly correlated with the number of panicles per plant and yield in both seasons. This observation suggests that severity of root galling may have a direct effect on yield-contributing plant traits. In other crops, such as cotton and tomato, water-deficit stress symptoms after root-knot nematode infection have been documented, and these were due to root gall formation that resulted in the disruption of the root epidermis, cortical cells and xylem [59, 60].

While our study identified several QTLs related to nematode reproduction, plant growth and yield-contributing traits, and grain yield under nematode-infected conditions, the contribution of each QTL based on R^2^ value is low. This indicates that several of the identified QTLs need to be pyramided for enhanced resistance to or tolerance for *M. graminicola*. The knowledge on positive/negative interaction between such identified alleles is necessary to successfully pyramid alleles that impart resistance to or tolerance of *M. graminicola*. The feasibility of advance genotyping technique such as the genotyping by sequencing (GBS) will allow us to adequately cover the full chromosomal regions and identify the smaller QTLs region as well as genes linked to markers. The application of genomic selection facilitated by GBS and the available phenotypic data will allow the identification of superior recombinants and fast track the development of rice genotypes with improved resistance to and tolerance for *M. graminicola*.

Resistance and tolerance can be an effective management tool that improve crop yield in the presence of nematode population densities [15]. The availability of a completely sequenced rice genome and the advancement of DNA markers have opened the opportunity to map for quantitative trait loci associated to quantitative traits such as disease resistance and tolerance. Identification of consistently and partially resistant genotypes with combined tolerance and that have the genetic loci for resistance, tolerance and yield may offer a good starting point for an MAB program to improve resistance to and tolerance for *M. graminicola*. Identified markers linked to resistance and tolerance can speed up the long process of traditional breeding as well as the deployment of improved rice genotypes for farmers’ use.

## Conclusion

In this study, we identified a total of 12 QTLs for the two season study that were related to *M. graminicola* resistance and tolerance and other plant traits such as yield, percentage of filled grains per plant and fresh and dry root weight in chromosomes 4, 5, 7, 8, 9, 10, 11 and 12. Rice genotypes that have the QTLs (see Additional file 1: Table S1) and which are either resistant or partially resistant and tolerant were also selected. In addition to resistance and tolerance, these genotypes were high-yielding in both nematode-infested and non-infested conditions. These selected genotypes that have the favorable alleles for the mapped QTLs and the identified QTLs are vital information in designing MAB that develops or improves those high-yielding rice genotypes susceptible to *M. graminicola* infection.

## Additional file

Additional file 1: **Table S1.** QTLs, yield and yield reduction of selected resistant and tolerant genotypes in two seasons. (DOCX 29 kb)

## Abbreviations

CIM: Composite interval mapping
CTAB: Cetylrimethyl ammonium bromide
DNA: Deoxyribonucleic acid
DRWT: Dry root weight
FG: Filled grains
FRWT: Fresh root weight
g: Gram
GYLD: Grain yield
J2: Second-stage juvenile
J2GRT: J2 per gram of roots
J2RS: J2 per root system
LOD: Logarithm of odds
MAB: Marker-assisted breeding
NPK: Nitrogen, phosphorous, potassium
QTL: Quantitative trait loci
R^2^: Percent phenotypic variance
RG: Root galling
RIL: Recombinant inbred line
SMR: Single marker regression
SNP: Single nucleotide polymorphism
SSR: Simple sequence repeats
YR: Yield reduction
