# Supplementary material for: QTL mapping for resistance to and tolerance for the rice root-knot nematode, Meloidogyne graminicola
Source: BMC Genet. 2018 Aug 6;19:53. doi: 10.1186/s12863-018-0656-1 (PMC6080554; doi:10.1186/s12863-018-0656-1)
Supplement: Supplementary file 1 — Table S1. QTLs, yield and yield reduction of selected resistant and tolerant genotypes in two seasons. (DOCX 29 kb) [file 12863_2018_656_MOESM1_ESM.docx]

Table S1. QTLs, yield and yield reduction of selected resistant and tolerant genotypes in two seasons.

| **Designation** | **QTLs** | **Reaction to nematode infection** | | **Yield per plant (g) nematode infested** | | **Yield per plant (g)**  **non-infested)** | | **Yield reduction (%)** | |
| --- | --- | --- | --- | --- | --- | --- | --- | --- | --- |
|  |  | **1^st^ season** | **2^nd^ season** | **1^st^ season** | **2^nd^ season** | **1^st^ season** | **2^nd^ season** | **1^st^ season** | **2^nd^ season** |
| IR 97152-B-47 | *qMGR_7.1_* | Resistant | Resistant | 16.0 | 14.5 | 16.5 | 15.0 | 3 | 4 |
|  | *qGYLD_10.1_* |  |  |  |  |  |  |  |  |
|  | *qFG_11.1_* |  |  |  |  |  |  |  |  |
| IR 97152-B-61 | *qMGR_9.1_* | Resistant | Resistant | 17.0 | 14.1 | 18.5 | 15.6 | 8 | 10 |
|  | *qMGR_7.1_* |  |  |  |  |  |  |  |  |
|  | *qYR_5.1_* |  |  |  |  |  |  |  |  |
|  | *qYR_11.1_* |  |  |  |  |  |  |  |  |
|  | *qGYLD_10.1_* |  |  |  |  |  |  |  |  |
|  | *qFG_11.1_* |  |  |  |  |  |  |  |  |
| IR 97152-B-89 | *qMGR_9.1_* | Resistant | Resistant | 18.1 | 19.2 | 20.0 | 20.0 | 10 | 4 |
|  | *qGYLD_10.1_* |  |  |  |  |  |  |  |  |
|  | *qFG_11.1_* |  |  |  |  |  |  |  |  |
|  | *qYR_11.1_* |  |  |  |  |  |  |  |  |
| IR 97152-B-113 | *qGR_8.1_* | Resistant | Resistant | 13.9 | 10.8 | 14.3 | 11.7 | 3 | 8 |
|  | *qGR_4.1_* |  |  |  |  |  |  |  |  |
|  | *qGYLD_10.1_* |  |  |  |  |  |  |  |  |
|  | *qFG_11.1_* |  |  |  |  |  |  |  |  |
| IR 97152-B-100 | *qMGR_5.1_* | Partially resistant | Partially resistant | 12.7 | 10.9 | 13.8 | 11.2 | 8 | 2 |
|  | *qYR_5.1_* |  |  |  |  |  |  |  |  |
|  | *qGYLD_10.1_* |  |  |  |  |  |  |  |  |

Table S1. Continuation.

| **Designation** | **QTLs** | **Reaction to nematode infection** | | **Yield per plant (g, nematode infested)** | | **Yield per plant (g, non-infested)** | | **Yield reduction (%)** | |
| --- | --- | --- | --- | --- | --- | --- | --- | --- | --- |
|  |  | **1^st^ season** | **2^nd^ season** | **1^st^ season** | **2^nd^ season** | **1^st^ season** | **2^nd^ season** | **1^st^ season** | **2^nd^ season** |
|  | *qFG_11.1_* |  |  |  |  |  |  |  |  |
| IR 97152-B-212 | *qMGR_9.1_* | Partially resistant | Partially resistant | 12.7 | 15.6 | 13.6 | 16.7 | 6 | 7 |
|  | *qMGR_7.1_* |  |  |  |  |  |  |  |  |
|  | *qYR_11.1_* |  |  |  |  |  |  |  |  |
|  | *qGYLD_10.1_* |  |  |  |  |  |  |  |  |
|  | *qFG_11.1_* |  |  |  |  |  |  |  |  |
